# Considerations for the clinical use of teplizumab in stage 2 Type 1 diabetes: A Consensus Statement from the British Society of Paediatric Endocrinology and Diabetes (BSPED) and the Association of British Clinical Diabetologists (ABCD)

**DOI:** 10.1111/dme.70329

**Published:** 2026-04-29

**Authors:** Renuka P. Dias, M. Loredana Marcovecchio, Ruben H. Willemsen, Fiona Campbell, Ahmad Ashour, Philip Newland‐Jones, Jennifer Cooper, Parth Narendran, Neil P. Wright

**Affiliations:** ^1^ Department of Paediatric Diabetes and Endocrinology Birmingham Women's and Children's NHS Foundation Trust Birmingham UK; ^2^ Department of Applied Health Sciences University of Birmingham Birmingham UK; ^3^ Department of Paediatrics University of Cambridge Cambridge UK; ^4^ Department of Paediatric Endocrinology and Diabetes Cambridge University Hospitals NHS Foundation Trust Cambridge UK; ^5^ Department of Paediatric Diabetes and Endocrinology The Royal London Children's Hospital, Barts Health NHS Trust London UK; ^6^ Department of Paediatrics Leeds Teaching Hospitals NHS Trust Leeds UK; ^7^ Department of Pharmacy The Royal London Children's Hospital, Barts Health NHS Trust London UK; ^8^ Diabetes and Endocrinology Service University Hospitals Southampton NHS Foundation Trust Southampton UK; ^9^ School of Human Development and Health University of Southampton Southampton UK; ^10^ Clinical Research Facility Birmingham Women's and Children's NHS Foundation Trust Birmingham UK; ^11^ Department of Diabetes Queen Elizabeth Hospital Birmingham UK; ^12^ Department of Immunology and Immunotherapy University of Birmingham Birmingham UK; ^13^ Department of Paediatric Endocrinology & Diabetes Sheffield Children's Hospital Sheffield UK

**Keywords:** ABCD, BSPED, diabetes prevention, disease modifying therapy, early stage 2 Type 1 diabetes, teplizumab

## Abstract

**Introduction:**

In 2025, the Medicines and Healthcare products Regulatory Agency (MHRA) approved the use of teplizumab (a monoclonal anti‐CD3 antibody) to delay progression from Stage 2 to Stage 3 Type 1 diabetes in the UK.

**Methods:**

To address the need for clear guidance on managing patients eligible for teplizumab therapy, the British Society of Paediatric Endocrinology and Diabetes (BSPED) Type 1 diabetes Special Interest Group (SIG) and the Association of British Clinical Diabetologists (ABCD) assembled a group to review the clinical trial data and develop expert consensus guidelines on the approach to teplizumab infusions.

**Conclusion:**

Here, we present recommendations on all aspects of teplizumab administration with evidence base where available. We highlight the safety considerations in individual's selection, screening, monitoring and treatment of potential common side effects.

## 1 INTRODUCTION

Type 1 diabetes is a chronic disease caused by immune‐mediated destruction of the pancreatic beta cells, which ultimately leads to the requirement for lifelong exogenous insulin replacement.^1^ The development of Type 1 diabetes occurs progressively over months and years before individuals present clinically with overt hyperglycaemia and classic osmotic symptoms, now termed stage 3 Type 1 diabetes.^2^

Given that Type 1 diabetes can be detected for many years before clinical symptoms emerge (Stage 3), a new classification of this prodrome of ‘early‐stage Type 1 diabetes’ has been developed with different stages.^2^ There is an initial phase characterised by the presence of multiple islet antibodies associated with normoglycaemia (Stage 1), followed by dysglycaemia (Stage 2) and finally by overt hyperglycaemia with or without symptoms (Stage 3).^2^, ^3^ These stages have now been assigned both SNOMED and ICD‐10 clinical codes, important levers to support health resource allocation^4^ (see Table S1). It is important to note with the data in Table S1, that progression risk data is based on highly selected cohorts with a first‐degree relative or with a high Type 1 Diabetes genetic risk score.

Once individuals have two or more islet antibodies (IAb), with or without dysglycaemia (i.e., Stage 1 or Stage 2 Type 1 diabetes), the natural history is such that they will almost certainly develop Stage 3, or symptomatic insulin‐requiring Type 1 diabetes, unless the autoimmune process can be modified or interrupted.^5^ Several drugs have demonstrated the potential to modulate the immune attack and preserve beta‐cell function.^6^ This approach offers the exciting possibility of preventing Stage 3 Type 1 diabetes and represents a paradigm shift towards focusing on moderating and halting the autoimmune attack rather than relying on exogenous insulin replacement as a first‐line therapy. Teplizumab is the first of several disease‐modifying therapies aimed at delaying progression to Stage 3 Type 1 diabetes to enter clinical practice.^7^, ^8^, ^9^

There are clear benefits to delaying the onset of Stage 3 Type 1 diabetes and related insulin therapy, including better glycaemic outcomes in at least the first five years post‐diagnosis^10^, ^11^; fewer hospital visits and burden associated with insulin therapy, including risk of hypoglycaemia.^12^ In addition, even a modest delay in the onset of clinical diabetes is likely to translate into significant improvement in life expectancy and reduced complications, particularly in younger individuals.^13^ Epidemiological data indicate that a diagnosis of clinical Type 1 diabetes before 10 years of age leads to a striking loss of 17.7 life‐years for women and 14.2 life‐years for men.^13^ This mortality data is of course based on historical cohort data, which does not take into account recent advances in diabetes technology, which have significantly improved HbA1c outcomes.^14^ Adolescents with Type 1 diabetes are a particularly vulnerable population, given that glycaemic outcomes in this age group remain suboptimal, even when diabetes technologies are implemented.^15^, ^16^ Follow‐up studies from the DCCT and EDIC cohorts have shown that even brief periods of improved diabetes management can lead to long‐lasting benefits, evident up to 30 years later.^17^ Furthermore, emerging evidence indicates that preservation of endogenous β‐cell function exerts sustained favorable effects on vascular outcomes, underscoring the potential importance of early therapeutic interventions aimed at mitigating β‐cell loss.^18^

## 2 TEPLIZUMAB

In 2022, the United States Food and Drug Administration (FDA) approved teplizumab for people with Stage 2 Type 1 diabetes from the age of 8 years. This represented a first‐in‐class license for a disease‐modifying treatment for Type 1 diabetes. It has now (in 2025) also been approved for use in Stage 2 Type 1 diabetes in Canada and the United Arab Emirates. In August 2025, the Medicines and Healthcare products Regulatory Agency (MHRA) approved teplizumab for use in Stage 2 Type 1 diabetes in the United Kingdom.^19^ In 2026, the EMA also approved Teplziumab in Stage 2 Type 1 diabetes. Currently, teplizumab has not been approved for use in the NHS by the National Institute for Clinical Excellence in the UK, with a decision pending in 2026. An NHS England Task & Finish group is currently exploring the implementation pathways and will conclude once the recommendations of the NICE technology appraisal are available. It is anticipated this would be funded through local integrated care boards (ICBs) rather than a funding variation in line with decentralisation of health funding linked to the 10‐year plan. Therefore, in the UK, we advocate early discussions by providers with their local ICBs (or equivalent) to support equitable implementation pathways based on local infrastructure.

### 2.1 Mechanism of action

Teplizumab is a humanised anti‐CD3 monoclonal antibody, which mitigates the auto‐immune destruction of pancreatic beta cells. It is immunomodulatory rather than immunosuppressive.^20^, ^21^

The CD3 complex provides the essential signal‐transducing elements of the T cell receptor, with teplizumab specifically targeting the ε chain of CD3, thereby modulating TCR signalling.^22^, ^23^, ^24^ Although its exact mechanisms of action are not completely understood, it is thought that teplizumab modulates the immune response through various effects on T cell subpopulations, including increases in specific CD8+ subtypes and partial exhaustion of CD8+ T cells, potentially resulting in reduced islet auto‐activity by rebalancing effector and regulatory T‐cells.^20^, ^21^ Teplizumab is typically given as a single treatment course of consecutive daily infusions over 14 days for Stage 2 Type 1 diabetes.^25^

### 2.2 Efficacy

Several trials have explored the safety and efficacy of teplizumab in Stage 2 and 3 Type 1 diabetes,^26^, ^27^, ^28^, ^29^ recently summarised by Herold et al.^7^

#### 2.2.1 Stage 2 Type 1 diabetes

The landmark trial of teplizumab in Stage 2, which led to FDA approval in 2022,^30^ was the TrialNet Teplizumab Prevention Study (TN10).^25^ This randomised double‐blind placebo‐controlled trial showed that a single 14‐day course of teplizumab delayed the median time to progression to Stage 3 Type 1 diabetes by approximately 24 months.^25^ An extended (unblinded) analysis of participants treated with teplizumab (*n* = 44) vs. placebo (*n* = 32) showed that there were further benefits, with the median time to progression delayed by 32 months, with 50% of participants given Teplizumab remaining disease‐free for over 5 years compared to 22% of controls.^21^, ^22^

#### 2.2.2 Stage 3 Type 1 diabetes

The first randomised controlled trial in Stage 3 Type 1 diabetes was published in 2002.^31^ Since then, teplizumab has been investigated in several trials, predominantly in newly diagnosed Stage 3 Type 1 diabetes.^7^ The most recent PROTECT study showed evidence of improved beta cell preservation, as assessed by the area under the curve of stimulated C‐peptide response during a mixed meal tolerance test at 12 months.^32^ Teplizumab is not yet approved for Stage 3 Type 1 diabetes, pending further efficacy data from larger clinical trials (βETA PRESERVE, NCT07088068).

These consensus guidelines focus on the practical aspects of Teplizumab, both to support decision making for eligibility and assessing adverse events during administration, once patients are identified as multiple IAb positive.

Currently, there is no plan for a national clinical screening programme for early‐stage Type 1 Diabetes in the UK. However, IAb screening is occurring outside of research programmes, with more than 50% of individuals currently in clinical care with early‐stage Type 1 diabetes identified outside of research.^33^ It is important that clinicians are able to undertake appropriate IAb screening in their clinical practice.

Additional supporting materials are being developed by the BSPED, ABCD and the patient‐led Digibete website to support starting conversations about antibody testing for healthcare professionals and to provide information to support patients and their families' decision‐making regarding screening.

## 3 METHODS

A UK‐based working group of 9 experts across the disciplines of paediatric and adult diabetology, pharmacy and nursing was assembled, and iterative agreement was reached on 13 statements of recommendation. The recommendations were then endorsed by the following learned societies: British Society for Paediatric Endocrinology and Diabetes (BSPED), Association of British Clinical Diabetologists (ABCD).

We present the list of recommendations for easy reference as Table 1.

**TABLE 1:** Complete list of recommendations.

Recommendation 1	Confirmation of Stage 2 Type 1 Diabetes requires evidence of dysglycaemia based on at least two abnormal glucose metrics at a single time point or the same abnormal metric confirmed at two separate time points between 3 and 12 months apart (as per Table S1)
Recommendation 2a	Individuals should be up to date with all appropriate vaccines as per the UK Green Book schedule, including influenza (https://www.gov.uk/government/collections/immunisation‐against‐infectious‐disease‐the‐green‐book#the‐green‐book)
Recommendation 2b	Live vaccines should be avoided within 8 weeks prior to receiving teplizumab and for 12 months after teplizumab infusion mRNA and inactivated vaccines are not recommended within 2 weeks prior to starting teplizumab and for 6 weeks post teplizumab infusion
Recommendation 3	Screening for active viral infections including Cytomegalovirus (CMV) and Epstein‐Barr virus (EBV) should be undertaken less than 4 weeks before the infusion start date As teplizumab has an immunomodulatory action, additional testing for Tubercolosis, HIV, Hepatitis B and C should be part of the screening bloods to determine suitability for teplizumab (see Table 2) Results should be interpreted in the context of clinical features (both at screening and during infusion)
Recommendation 4	Teplizumab treatment should not given to women who are pregnant or within 30 days prior to planned pregnancy. Breastfeeding should not be continued during Teplizumab administration (see also SMPC)
Recommendation 5	Centres delivering teplizumab should have trained staff in aseptic preparation, administration and adverse reaction management
Recommendation 6	If a planned infusion is missed, resume dosing on consecutive days to complete the 14‐day course. Do not administer two doses on the same day
Recommendation 7a	See also Tables 4 and S2 *Before teplizumab initiation* Neutrophil count should be >1.5 × 10^9^/L Lymphocyte count should be >1.0 × 10^9^/L Platelet count should be >150 × 10^9^/L Haemoglobin should be >100 g/L
Recommendation 7b	See also Tables 4 and S2 *During teplizumab infusion* If neutrophil count falls to <0.5 × 10^9^/L, *use clinical discretion* and consider pausing the infusion for that day. If there is no recovery trend by 7 days despite pausing, it is recommended to discontinue the infusion If lymphocyte count falls to <0.5 × 10^9^/L, *use clinical discretion* and consider pausing the infusion for that day. If there is no recovery trend by 7 days despite pausing, it is recommended to discontinue the infusion course If the platelet count falls to <50 × 10^9^/L, a pause in infusions is recommended. If there is no recovery after 7 days despite pausing, it is recommended to discontinue the infusion If the haemoglobin falls below 100 g/L, infusions are paused and discontinued if levels are less than 85 g/L
Recommendation 8a	See also Tables 4 and S2 *Before teplizumab initiation* Alanine transaminase (ALT) or aspartate transaminase (AST) should be <2× upper limit of normal Bilirubin should be <1.5× upper limit of normal
Recommendation 8b	See also Tables 4 and S2 *During teplizumab infusion* If either ALT or AST is ≥3× the upper limit of normal, pause the infusion for that day and stop the infusion if there has been no recovery after 3 days If either ALT or AST is ≥5× the upper limit normal, stop the infusion course If the AST or ALT is ≥3× the upper limit of normal AND the bilirubin is ≥2× the upper limit of normal, stop the infusion course If the Bilirubin is ≥2 times the upper limit of normal, pause the infusion for that day and discontinue the infusion if there is no recovery after 3 days If the bilirubin ≥3× the upper limit of normal, stop the infusion course
Recommendation 9:	During the phase of white cell fall, it is recommended that individuals wear a face mask when in public places including school, work offices and on public transport. In addition, standard infection control measures should be used including handwashing
Recommendation 10a	Centres delivering teplizumab should have: A clear escalation plan to high‐dependency or intensive care if required Ready access to haematology/immunology and critical care support
Recommendation 10b	To mitigate the risk of Cytokine Release Syndrome (CRS): Administration of anti‐pyretic (paracetamol or non‐steroidal anti‐inflammatory drugs (NSAID)) and antihistamine (chlorpheniramine) should be part of pre‐medication Consider giving paracetamol/NSAID and antihistamine regularly in the first 5 days of treatment and as required on subsequent infusion days Consider giving an anti‐emetic (ondansetron) as required Services delivering teplizumab must be prepared to recognise and treat Cytokine Release Syndrome (CRS) promptly
Recommendation 11a	Close glucose monitoring needs to be undertaken throughout the period of teplizumab administration
Recommendation 11b	If well tolerated, teplizumab infusion should be continued with insulin treatment for hyperglycaemia as needed
Recommendation 12	Home infusion should be avoided in the first week of dose escalation as this is when adverse events are most likely to occur
Recommendation 13	Monitoring of individuals who have received teplizumab should be as per most up‐to‐date national or local consensus guidelines (may vary between adults and children)

## 4 PRACTICAL CONSIDERATIONS

### 4.1 Selection of individuals to receive treatment

The current license for teplizumab is for individuals 8 years of age or older diagnosed with Stage 2 Type 1 diabetes. The FDA has just (April 2026) approved the use of teplizumab in children over 1 year with Stage 2 Type 1 Diabetes. The PETITE‐T1D trial (teplizumab in Paediatric Stage 2 Type 1 diabetes, NCT05757713) investigated the efficacy and safety in children younger than 8 years reporting Teplizumab is well tolerated in the young age group with similar adverse event profile.^34^

Although teplizumab is licensed for older age, the natural history of Type 1 diabetes in adults, and therefore the confidence with which we can estimate the benefits of this therapy in these older age groups, remain largely unknown. We suggest that, for now, teplizumab has proven benefit in adults up to the age of 45, but may also benefit people in older groups. We suggest this age because this was the age range in the TN10 study.^25^ Restricting to younger adults may also avoid some of the issues with the accurate classification of diabetes when screening older adults.^35^

In addition, patient views (adults with or caregivers of young people with early‐stage Type 1 Diabetes) have been explored both in terms of being offered therapy and the prospect of therapy being available.^36^, ^37^

### 4.2 Staging criteria

International and national consensus guidelines for monitoring of individuals with early‐stage Type 1 diabetes for progression have recently been published and should also be referred to identify people at stage 2 who might be eligible for teplizumab.^3^, ^38^

Individuals eligible for teplizumab should be classified as Stage 2 based on seropositivity for 2 or more IAb *and* dysglycaemia, defined as meeting at least two different criteria for dysglycaemia at a single time point or meeting the same single criterion at two time points between 3 and 12 months apart (see Table S1).^3^

There is fluidity between early stages of Type 1 diabetes, particularly based on single glycaemic metrics, and these criteria ensure that patients have not reverted to Stage 1 or moved into Stage 3 at the time of eligibility.^39^ There should be no diagnostic uncertainty regarding possible monogenic diabetes or Type 2 diabetes.

The TN10 trial was undertaken in individuals at high risk (family history of Type 1 diabetes) and confirmed dysglycaemia on oral glucose tolerance test (OGTT).^2^, ^25^, ^33^ The staging of early‐stage Type 1 diabetes now includes HbA1c in addition to OGTT criteria.^2^, ^40^

There is currently no research evidence for the use of teplizumab for individuals who have reverted from Stage 3 (with or without insulin treatment) to persistent Stage 2. This may change with future research evidence. Here, we suggest that individuals who are in Stage 2 for three or more months (having been in Stage 3 for no more than 6 months with or without insulin previously) should be considered for treatment with teplizumab.

There is some evidence that continuous glucose monitoring (CGM) data trends may be able to define dysglycaemia, particularly in terms of risk of progression to Stage 3.^41^, ^42^ This may improve patient acceptance in real‐world settings. However, a recent meta‐analysis suggested that further work is needed to explore the utility of CGM for staging.^42^

### 4.3 Vaccination

As per license and administration, live vaccines should be avoided within 8 weeks prior to receiving teplizumab and for 12 months after teplizumab infusion.^30^

mRNA and inactivated vaccines are not recommended within 2 weeks prior to starting teplizumab and for 6 weeks post teplizumab infusion.^30^

### 4.4 Pre‐treatment screening

There are several additional criteria that must be met as part of the pre‐treatment screening for teplizumab to ensure safety, including no active serious or chronic active infections (other than localised skin infections).

### 4.5 Negative pregnancy test (for females of reproductive age)

There is no safety data available for use of teplizumab for women who are pregnant, actively considering starting a family or breastfeeding. Pregnancy tests should be conducted before infusions, and reliable contraception methods should be discussed with males and females of childbearing age. Endogenous maternal IgG and monoclonal antibodies are known to be excreted in human milk. Breastfeeding should not be continued during teplizumab administration. See also the Summary of Product Characteristics (SMPC) about breastfeeding and Teplizumab administration postpartum.

As part of the eligibility assessment for teplizumab, screening bloods listed in Table 2 is recommended.

**TABLE 2:** Pre‐treatment screening investigations and thresholds for teplizumab initiation.

Test	Requirement
Full blood count	Hb >100 g/L
	Platelets >150 × 10^9^/L
	Lymphocyte count >1.0 × 10^9^/L
	Neutrophil count >1.5 × 10^9^/L
Liver function test	ALT and AST <2× upper limit of normal (ULN)
	Bilirubin <1.5 ULN
EBV PCR (IgM)	Negative Plus no clinical evidence of acute infection
CMV PCR (IgM)	Negative Plus no clinical evidence of acute infection
TB (ideally Quantiferon Gold)	Negative Plus no clinical evidence of acute infection
HIV, Hepatitis B and Hepatitis C serology	Negative
Pregnancy test (in females of childbearing age)	Negative

Abbreviations: CMV, cytomegalovirus; EBV, Epstein–Barr virus; HIV, human immunodeficiency virus; TB, tuberculosis; ULN, upper limit of the normal range.

### 4.6 Equipment

Safe delivery of teplizumab requires access to appropriate IV administration equipment, preparation facilities and monitoring resources. (See Appendix S1 for additional preparation/storage and adminstration instructions).

### 4.7 IV access options

IV access will be required for the 14‐day infusion and for blood tests. There are several options:

- Peripheral venous cannula (PVC). Some centres allow patients to go home with a PVC in place; local policies should be followed.
- Midline catheter may be considered in individuals with poor peripheral access or anticipated repeated cannulation during the 14‐day course.
- Lines should be checked for patency and evidence of infection/skin irritation before each infusion.

### 4.8 PVC infusion materials

Teplizumab must be prepared and administered in line with the UK SmPC, including use of PVC infusion materials (PVC infusion bag/tubing) and the specified dilution method. If alternative (non‐PVC) materials are proposed locally, this should only occur where compatibility data are available and documented, with medicines governance approval. Infusion should be started within 2 h of preparation and discarded if not administered within 4 h (SmPC).

### 4.9 Preparation

Close liaison and support from a hospital pharmacist is suggested to avoid inadvertent wastage of the product, including ensuring fridge fail safes are in place.

Teplizumab comes as a sterile, preservative‐free, clear and colourless solution, which needs to be diluted in saline prior to use. This is done as a standard aseptic technique and can be done in wards.

### 4.10 Dosing

Teplizumab is given as a single 14‐day consecutive infusion as per efficacy data from clinical trials for Stage 2 Type 1 diabetes.^25^ This includes an initial 5‐day period of dose escalation and then days 6–14 at dose maintenance (see Table 3). Dose interruption effects are currently unknown. However, doses may have to be withheld in specific situations (see Table 2 and a section on safety profile for withholding criteria). Note individuals with a BSA >1.94 m^2^ may need 2 vials per dose from D5.

**TABLE 3:** Schedule of investigations during infusion.

Day	Pre infusion	1	2	3	4	5	6	7	8	9	10	11	12	13	14
FBC	X	X		X		X			X						X
U&E	X	X													
LFT (inc AST/ALT and Bilirubin)	X	X		X		X			X						X
Pregnancy test^a^		X													
Viral serology	X														
TB test	X														
Infusion dose (mcg/m^2^)		65	125	250	500	1030	1030	1030	1030	1030	1030	1030	1030	1030	1030

Abbreviations: ALT, alanine aminotransferase; AST, aspartate aminotransferase; FBC, full blood count; LFT, liver function tests; TB test, tuberculosis test; U&E, urea and electrolytes.

^a^ Females of childbearing age.

### 4.11 Administration

Although the risk of severe cytokine release syndrome (CRS) is rare, it is important to ensure that there is adequate escalation in place including a high dependency and/or intensive care unit available as well as haematology/immunology support for management.

The named (prescribing) or on‐call diabetes consultant should be responsible for reviewing laboratory data and clinically evaluating individuals for new/ongoing symptoms. Regular investigations should be undertaken throughout the 14‐day course of treatment to ensure any adverse events are detected and managed (Table 3).

### 4.12 Safety profile (see Table 4)

**TABLE 4:** Managing other adverse events with teplizumab.

Potential adverse effect	Incidence in Integrated Safety Analysis (*n* = 1018) % teplizumab (% placebo)	Action
Haematological		
Lymphopenia	79.9 (16.7)	If the lymphocyte count falls to <0.5× × 10^9^/L and shows no recovery within 7 days, teplizumab should be discontinued
Neutropenia	39.6 (21.6)	If the neutrophil count falls to <0.5× × 10^9^/L and shows no recovery within 7 days, teplizumab should be discontinued Consider pausing if <0.5× 10^9^/L, depending on clinical evaluation
Anaemia	28.8 (22.4)	Hb <100 g/L, pause infusion and wait for anaemia to recover Hb <85 g/L, discontinue treatment with teplizumab
Thrombocytopenia	21.7 (9.8)	Platelets <50 × 10^9^/L, pause infusion until count increased If there is no increase after 7 days despite pausing, discontinue the infusion
Infection		
Mild Infection eg Viral URTI	19.0 (17.6)	Treat symptomatically & consider pausing infusion depending on clinical evaluation (e.g., appears unwell, fever, abnormal CRP)
EBV & CMV	EBV 2.3 (4.1)	If positive PCR on screening, do not treat with teplizumab No need to monitor during infusions, if no viraemia prior to treatment, unless symptomatic
Liver function tests		
Bilirubin		2× Upper limit normal, pause infusions to check recovery. Discontinue infusion if no recovery within 3 days. 3× Upper limit normal, discontinue infusion
AST	28.1 (20.4)	3× Upper limit normal, pause infusions to check recovery. Discontinue infusion if no recovery within 3 days 5× Upper limit normal, discontinue infusion
ALT	26.5 (11.4)	3× Upper limit normal, pause infusions to check recovery. Discontinue infusion, if no recovery within 3 days 5× Upper limit normal, discontinue infusion
Cytokine Release Syndrome (CRS) 5.8 (1.2)		
Fever	23.8 (16.7)	Pause infusion if febrile. Treat with anti‐pyretics If fever resolves within <48 h and clinically well, continue infusion course If fever persists >48 h, discontinue infusions and consider additional treatment (e.g., steroids)
Mild (88% of all CRS)		Pause infusion and consider additional treatment (e.g., steroids) if unwell. Rash may be a feature of CRS, but not all rashes represent CRS If persists for >2 days, discontinue infusion
Severe		Discontinue infusions and consider additional treatment, e.g., steroids, Toclizumab (local PICU/ICU protocols to be applied)
Others		
Anaphylaxis (Rare acute IgE mediated reaction)		Discontinue infusions Fluids and vasopressors for hypotension if required Consider supportive treatment as per local protocols
Rash	34.5 (10.2)	Manage symptomatically Can continue infusions if clinically well Consider whether to pause/stop if rash is likely associated with CRS

Abbreviations: CMV, cytomegalovirus, CRS, cytokine release syndrome; EBV, Epstein–Barr virus; URTI, upper respiratory tract infection.

Over one thousand people have received teplizumab under the umbrella of a clinical trial and an increasing number in clinical practice in the United States and United Kingdom.^36^ Most data on the safety profile and adverse events are drawn from an integrated safety analysis published in 2023 by Herold et al. In total, 1018 people with Stage 2 or Stage 3 Type 1 diabetes across 6 clinical trials were assessed, 75.9% assigned teplizumab, 24.1% assigned to placebo.^7^ In addition, the PROTECT study included 217 individuals who received teplizumab.^32^

Potential adverse effects from teplizumab broadly fall into the following categories:

1. Haematological abnormalities
2. Risk of infection
3. Transaminitis
4. CRS
5. Rash

### 4.13 Haematological abnormalities

#### 4.13.1 White cell abnormalities

**FIGURE 1:**
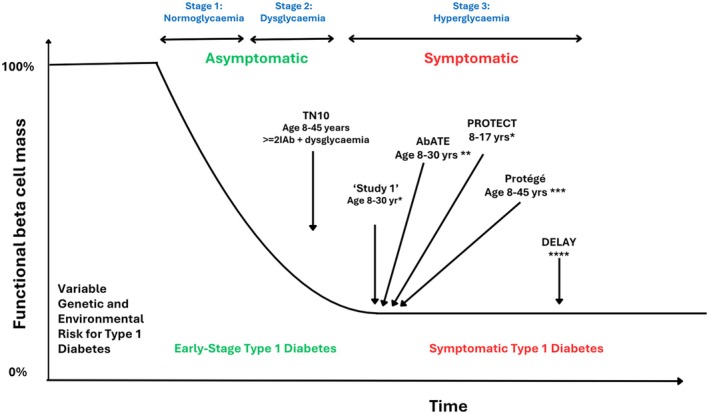
Summary of studies investigating teplizumab in Type 1 diabetes. *Within 6 weeks of Stage 3 diagnosis **within 8 weeks of Stage 3 diagnosis ***within 12 weeks of Stage 3 diagnosis ****within 4–12 months of Stage 3 diagnosis.

**FIGURE 2:**
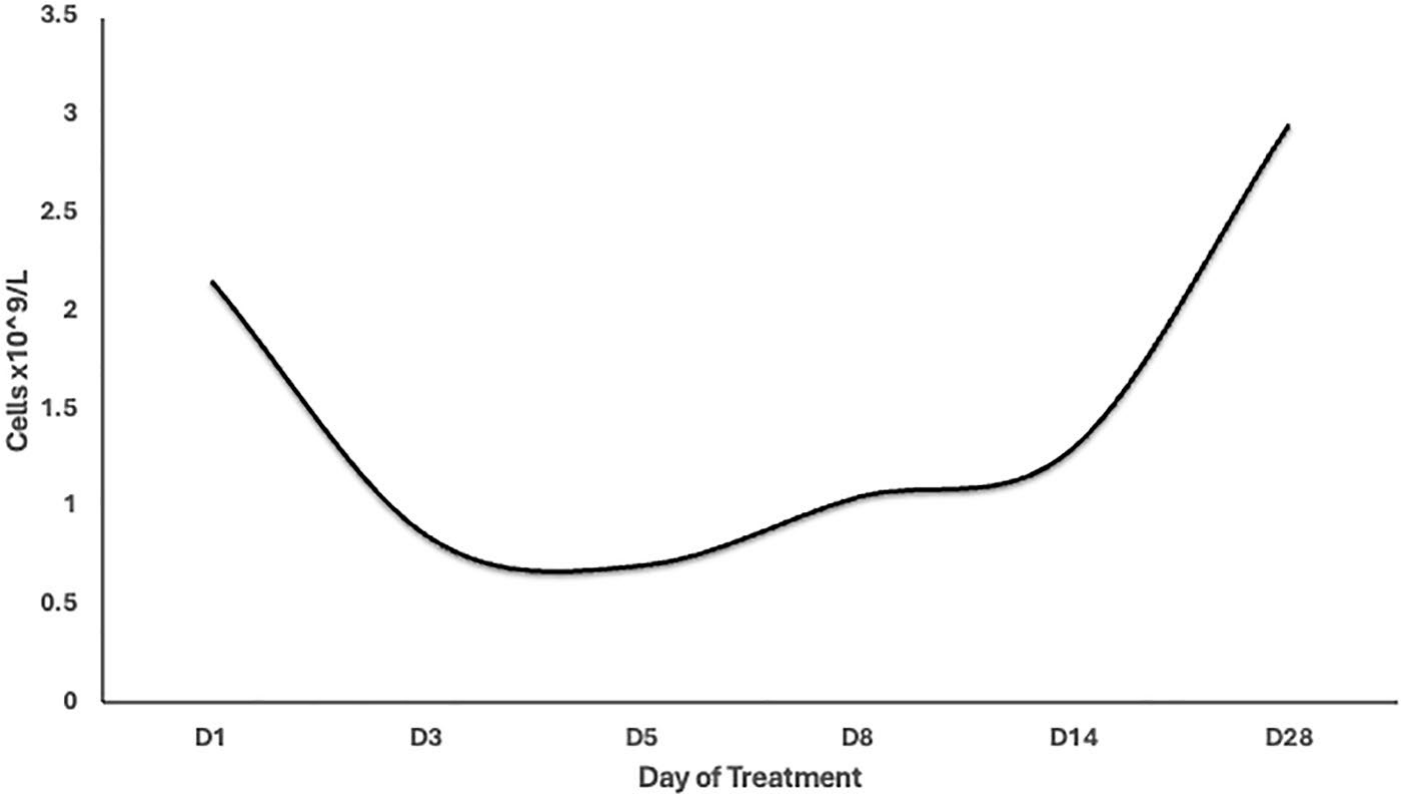
Graphical representation of the changes in lymphocyte count over the treatment course with teplizumab (based on UK clinical data and TN10 trial data).

Lymphopenia, leukopenia and neutropenia are extremely common and are to be anticipated during infusion of teplizumab. In the integrated safety analysis, 80% of participants experienced lymphopenia, 60% leukopenia and 40% neutropenia. In almost all cases, these abnormalities were self‐limiting with cell counts returning to normal by 28 days. Figures 1 and 2 illustrate the change in lymphocyte count with the nadir typically occurring between days 4 and 5, with white counts increasing during the remainder of the 9–10 days of the infusion course. It is believed that the white count falls due to margination of white cells outside the circulation, particularly to the gastrointestinal tract, rather than true white cell depletion.^43^ Table 4 outlines the pause/stop criteria for teplizumab for haematological abnormalities. In addition, Table S2 provides an overview of the pause/stop criteria in other clinical trials and protocols.

#### 4.13.2 Platelet abnormalities

Thrombocytopenia has also been noted more frequently in the teplizumab than placebo group (22% versus 10%).^32^

#### 4.13.3 Red cell abnormalities

Anaemia has also been noted more frequently in the teplizumab than the placebo group (27% versus 21%).^44^

Within the integrated safety analysis, 14.3% of individuals discontinued infusions during the planned course of treatment compared to 3.7% in the placebo group.^7^ These were mainly due to predefined laboratory abnormalities. Approximately 3% of those who discontinued treatment were due to neutropenia, thrombocytopenia, lymphopenia or anaemia.^7^

### 4.14 Infection risk

Whilst reductions in the white cell counts are frequently noted and in fact expected, there does not seem to be a significant risk of severe infections during teplizumab infusions. This is consistent with the concept that this is an immunomodulatory rather than immunosuppressive treatment. In the integrated safety analysis, infection rates were similar between the teplizumab and placebo groups (53% versus 52.3%).^7^ There was no increase in severe infections reported in the PROTECT study (0.5% grade 3 infection in the teplizumab arm vs. 1.8% in the Placebo arm).^32^ It is notable that the PROTECT study was conducted during the SARS CoV‐2 pandemic (2020–2022), and SARS CoV‐2 was reported in 22.6% of participants in the teplizumab and 23.4% in the placebo group, with none requiring hospital or antiviral treatment.^32^

In healthy individuals, CD8^+^ cytotoxic T cells suppress viral reactivation by recognizing and killing EBV‐infected cells. When anti‐CD3 therapy transiently suppresses or depletes T cells, this reduces immune surveillance against EBV, allowing the virus to reactivate from latency in B cells.

The potential for teplizumab to increase vulnerability to reactivation of previous EBV and CMV infections has been studied within the trials. This is particularly pertinent because of the risk of lymphoproliferative disorder associated with EBV. There have been no reported cases of malignancy or lymphoproliferative disorders associated with teplizumab. EBV reactivation was noted in 5.1% of teplizumab‐treated and 2.4% of control participants. In the integrated analysis over several years, new infections with EBV during the study period were more common in the placebo groups (2.3% teplizumab vs. 4.1% Placebo). EBV was detected in 3.2% of teplizumab versus 1.2% of placebo participants, typically several weeks after the infusion. Rates of new CMV infection were similar in both groups (0.6% teplizumab vs. 0.8% Placebo), and there was some evidence of reactivation of CMV in 0.5% of individuals. When EBV and CMV viraemia were detected, they were generally asymptomatic and resolved spontaneously.^7^

### 4.15 CRS

CRS is a potentially serious systemic inflammatory response caused by cytokine release following immunotherapy.^7^ It is typically mild and self‐limiting but can progress to significant haemodynamic and respiratory compromise requiring inotropic and ventilatory support. It encompasses a range of features, with its principal one being fever. A caveat to this is that fever can be impacted by pre‐medication. Therefore, in mild CRS, the constellation of symptoms is important to consider. Other manifestations can include mild nonspecific symptoms such as nausea, headache, myalgia and rash. In severe cases, hypotension and hypoxia can occur.^45^

CRS was noted in 5.8% of teplizumab‐treated versus 1.2% of placebo‐treated participants in the integrated safety analysis.^7^ Most events occurred within the first 3 to 5 days of the infusions and resolved spontaneously within 2 to 3 days from the onset. In most cases, CRS was mild (88%) or moderate in severity based on trial CTCAE grading.^7^

To mitigate the risk of CRS, premedication with an anti‐pyretic (paracetamol or non‐steroidal anti‐inflammatory drugs (NSAID, e.g., Ibuprofen)) and an antihistamine (e.g., chlorpheniramine maleate) is recommended. Trial protocols have recommended regular premedication for the first five days of the infusion, with continuation thereafter if clinically indicated.^22^

In severe CRS, hypotension may need support with intravenous fluids and potentially inotropes. In such cases, support from haematologists or intensivists within a high dependency setting is likely to be required. Treatment with glucocorticoids, mainly dexamethasone, has been shown to be effective in CRS.^45^ Additional therapies where initial treatment is not effective need to be discussed with local critical care and haematology/immunology teams. However, such events are expected to be rare and not reported in previous trials.^7^

Symptoms including fever, rash or malaise may develop outside of the post infusion monitoring period. Patients should be advised what potential adverse events might occur and the contact details of the hospital team (both in hours and out of hours) if they develop symptoms or are concerned. Healthcare providers should have the capacity to be contactable for patient queries post discharge and be able to admit patients either during the daycase admission or following discharge from the daycase if clinically indicated.

### 4.16 Liver function tests

Raised liver transaminases and rash may also be features of CRS, although they are not specific. Raised liver transaminases are common, occurring in approximately 25% of individuals receiving teplizumab, and this is the commonest reason for infusion discontinuation. Most individuals with elevated transaminases will not have CRS. However, among those with CRS, raised transaminases were noted in 56% of cases. Discontinuation of teplizumab due to raised transaminases occurred in 7.7% of patients.^7^ We have aligned with the PROTECT protocol for transaminitis pause criteria based on wider discussion with US colleagues with experience of treating >30 patients, but clinical discretion should be used throughout.^32^

### 4.17 Rash and other adverse effects

A rash was a common feature in the clinical trials of teplizumab, independently of CRS, and was observed in 34.5% of those treated with the active drug and 10.2% of those receiving placebo.^7^ This was typically non‐pruritic maculopapular in nature or peeling of hands and feet.

Headache and nausea were the commonest adverse events reported, occurring in 27.2% and 19.6% participants, respectively.^7^

Fatigue has been noted with teplizumab therapy and should be highlighted to adults who may be driving on treatment days.

### 4.18 Drug interactions

No clinically relevant CYP450‐mediated interactions are expected with teplizumab, consistent with its monoclonal antibody mechanism of action.^30^ Large integrated safety analyses also confirmed no unexpected interaction signals.^7^

## 5 OTHER ISSUES

### 5.1 Hyperglycaemia and progression to stage 3 during teplizumab infusion

In both the United Kingdom and United States, there have been cases of progression from Stage 2 to overt hyperglycaemia requiring insulin treatment during the administration of teplizumab. In most cases, this has been transient, with individuals reverting to Stage 2 or even Stage 1 post teplizumab without any ongoing need for insulin therapy (personal communication Narendran, Willemsen and Simmons).

It is anticipated that closer glucose monitoring is undertaken throughout the period of Teplizumab administration (CGM or capillary blood glucose), with at least once daily blood glucose checks pre‐infusion as the minimum. All patients should have access to a capillary blood glucose monitor and a ketone meter to perform additional testing outside of this if unwell/concerned. Health care providers may wish to use real‐time CGM. In all instances, treatment of hyperglycaemia with insulin should be as per local protocol/clinical discretion.

## 6 HOME INFUSION

At present, there is no published data on managing teplizumab as a home infusion. However, it is anticipated that as diabetes teams become more confident, home infusion therapy will develop depending on capacity. Home infusion is potentially more feasible in the second week (after dose escalation, due to less blood monitoring requirements and less likelihood of adverse reactions occurring once confidence and national experience with administration increase).^7^, ^32^ However, the risk of adverse events needs to be evaluated on an individual basis, and the default setting for infusion should be in hospital, particularly for the paediatric population. There needs to be a clear and robust standard operating procedure in place to ensure (a) prompt review of required blood investigations (e.g., white cell count and transaminases) and (b) prompt clinical evaluation of the patient if an adverse event occurs.

### 6.1 Monitoring/follow up

Individuals who have received teplizumab should still be monitored as per consensus guidelines for early‐stage Type 1 diabetes.^3^, ^38^

This should include

1. Ongoing education about signs and symptoms (4 T's – https://www.diabetes.org.uk/living‐with‐diabetes/life‐with‐diabetes/children‐and‐diabetes/symptoms) of Stage 3 Type 1 diabetes with 3‐monthly review.
2. Ongoing surveillance for rising trends in glucose metrics. This could be a capillary blood glucose (CBG) check 2 h after a carbohydrate meal, CGM metrics (CGM), 3–6 monthly HbA1c and/or annual OGTT depending on age and stage.^35^, ^38^
3. Signposting for appropriate research studies if eligible.

Insulin initiation should be undertaken with the advice of the diabetes team and based on patterns from regular CBG or CGM data and depending on the presence of symptoms.

## 7 CONCLUSIONS

Teplizumab is the first disease‐modifying therapy approved for delaying progression from Stage 2 to Stage 3 Type 1 diabetes. The above recommendations reflect expert consensus based on clinical experience and research trials delivering teplizumab. They intend to provide clear guidance for healthcare professionals in the UK. As real‐world experience grows, this initial guidance will need to be updated.

It is important that these recommendations are taken as guidance, and each centre needs to develop locally ratified protocols and implement training for staff, including the management of adverse reactions.

It is important that individuals receiving teplizumab have ongoing monitoring, as they will develop Stage 3 Type 1 diabetes without additional intervention to delay progression.

## FUNDING INFORMATION

No funding has been received for this work. R.P.D. is supported by National Institute for Health Research Award (Ref NIHR304587).

## CONFLICT OF INTEREST STATEMENT

RPD has received speaker fees from Sanofi and Sandoz. RPD, MLM, FC, NPW, PN and RHW have received honoraria from Sanofi (participation in advisory board for teplizumab). RHW has received consultancy fees from Sanofi (review of HCP and patient materials for teplizumab), conference attendance support from Sanofi and speaker fees from Insulet. PNJ has received speaker fees, honoraria and conference support from Sanofi, Eli Lilly and Abbott. JC has received consultancy fees from Sanofi. NPW has received conference support from Sanofi. PN has received speaker fees from Sanofi, Lilly, Abbott.

## Supporting information


Table S1:

Table S2:

Appendix S1:
