# Supplementary material for: Considerations for the clinical use of teplizumab in stage 2 Type 1 diabetes: A Consensus Statement from the British Society of Paediatric Endocrinology and Diabetes (BSPED) and the Association of British Clinical Diabetologists (ABCD)
Source: Diabet Med. 2026 Apr 29;43(7):e70329. doi: 10.1111/dme.70329 (PMC13257899; doi:10.1111/dme.70329)
Supplement: Supplementary file 1 — Table S1: [file DME-43-e70329-s003.docx]

**Supplementary Table 1: Staging of Type 1 diabetes** (adapted from (3))

|  | **Presymptomatic T1D** | | **Symptomatic T1D** |
| --- | --- | --- | --- |
|  | **Stage 1** | **Stage 2** | **Stage 3** |
| **IAb status** | ≥2 IAB positive | ≥2 IAb positive | NA |
| **Fasting Plasma Glucose** | <5.6 mmol/L | 5.6-6.9 mmol/L | ≥7.0 mmol/L |
| **120 min Plasma  Glucose in OGTT** | <7.8 mmol/L | 7.8-11.0mmol/L | ≥11.1 mmol/L |
| **HbA1c** | <39 mmol/mol (<5.7%) | 39-47 mmol/mol (5.7-6.5%)  or a ≥10% increase in HbA1c) | ≥ 48 mmol/mol  (≥6.5%) |
| **Interim Glucose (30/60/90 min values) in OGTT** | <11.1 mmol/L | ≥ 11.1 mmol/L | ≥11.1 mmol/L  Plus osmotic symptoms or HbA1c ≥48mmol/mol |
| **5-year rates of progression to insulin requirement** | 44% | 75% | Not applicable |
| **ICD 10 code** | E10A1 | E10A2 | E10.9 |
| **SNOMED** | 1290118005 | | 46625009 |

***Legend Supplementary table 1:*** *IAb: islet autoantibody; FPG: fasting plasma glucose; OGTT: Oral glucose tolerance test; HbA1c: glycated hemoglobin; ICD-10: International Classification of Diseases, 10th Revision; SNOMED: Systematized Nomenclature of Medicine*
