# Supplementary material for: Considerations for the clinical use of teplizumab in stage 2 Type 1 diabetes: A Consensus Statement from the British Society of Paediatric Endocrinology and Diabetes (BSPED) and the Association of British Clinical Diabetologists (ABCD)
Source: Diabet Med. 2026 Apr 29;43(7):e70329. doi: 10.1111/dme.70329 (PMC13257899; doi:10.1111/dme.70329)
Supplement: Supplementary file 2 — Table S2: [file DME-43-e70329-s001.docx]

**Supplementary Table 2: Pause/Stop criteria from US consensus, PROTECT, FDA and MHRA**(18,29,31,38)

|  | US Pediatric Endocrine Society Consensus | | PROTECT | | FDA | | TN10 | | MHRA | |
| --- | --- | --- | --- | --- | --- | --- | --- | --- | --- | --- |
| **Parameter** | **Stop criteria** | **Pause criteria** | **Stop criteria** | **Pause criteria** | **Stop criteria** | **Pause criteria** | **Stop criteria** | **Pause criteria** | **Stop criteria** | **Pause criteria** |
| **Liver** |  |  |  |  |  |  |  |  |  |  |
| Bilirubin | 3 x ULN | 1.5 x ULN | > 3x ULN,  or ALT/AST > 3x ULN and Bili > 2x ULN | > 2x ULN and < 3x ULN | > 3x ULN | NA | NA | Bilirubin >1.3 mg/dl on Day 1, ≥2.0 mg/dl on other | > 3xULN | NA |
|  |  |  |  |  |  |  |  | days. |  |  |
| AST | 5 X ULN | 2x ULN | > 5x ULN | > 3x ULN but <5 x ULN | > 5x ULN | NA | NA | AST level >2 times ULN on Day 1.  AST ≥3.0 times ULN on other days. | > 5xULN | NA |
| ALT | 5 X ULN | 2x ULN | > 5x ULN | > 3x ULN but <5x ULN | > 5x ULN | NA | NA | ALT or LDH ≥3.0 times ULN on other days. | > 5xULN | NA |
| **CRS** |  |  |  |  |  |  |  |  |  |  |
| Severe CRS | If continues > 2 days | If resolved within 2 days | Yes | Yes | consider stopping if severe CRS | consider pausing 1-2 days until recovered | NA | NA | > 2 days CRS | NA |
| **Haematological** |  |  |  |  |  |  |  |  |  |  |
| Lymphocytes | < 500 cells/μ l and no recovery within 7 days | NA | NA | NA | < 500 cells /μ l >= 7 days | NA | NA | NA | < 0.5x10^9^/l for > 7 days | NA |
| Neutrophils | < 500 cells/μl and no recovery within 7 days | < 500 cells/ μ l | NA | 500-1000 cells/ μl | NA | NA | NA | <1000 cells/μ l | NA | NA |
| Hb | < 8.5 g/L | < 10.0 g/L | NA | >8.5 but > 10.0 g/dL | NA | NA | NA | ≤ 8.5 g/dL or a drop in ≥2g/dL compared with prior to | NA | NA |
|  |  |  |  |  |  |  |  | infusion to a value < 10.0 g/dL |  |  |
|  |  |  |  |  |  |  |  |  |  |  |
| Platelet count | < 50,000/μl and no recovery by 7 days | < 50, 000/μ l | NA | >40,000 but<100,000 cells/ul | NA | NA | NA | < 140,000/μl on Day 1 and < 100,000 on other | NA | NA |
|  |  |  |  |  |  |  |  | days |  |  |
| **Miscellaneous** |  |  |  |  |  |  |  |  |  |  |
| **Missed doses for >3 days** | NA | NA | If drug paused for 2 consecutive days, consider stopping drug course | NA | NA | NA | NA | NA | NA | NA |
| **Hypersensitivity reaction** | Angioedema, serum sickness, bronchospasm | NA | NA | NA | Severe | NA | NA | NA | NA | NA |
| **Serious infections** | NA | NA | NA | NA | Stop | NA | NA | NA | Stop | NA |
| **New /reactivated EBV** | Likely if confirmed | NA | NA | NA | NA | NA | NA | NA | NA | NA |

***Legend supplementary table 2:*** *AST: aspartate aminotransferase; ALT: alanine aminotransferase; CRS: cytokine release syndrome; Severe CRS: grade ≥3 cytokine release syndrome; Hb: hemoglobin; EBV: Epstein–Barr virus*
