# Supplementary material for: Considerations for the clinical use of teplizumab in stage 2 Type 1 diabetes: A Consensus Statement from the British Society of Paediatric Endocrinology and Diabetes (BSPED) and the Association of British Clinical Diabetologists (ABCD)
Source: Diabet Med. 2026 Apr 29;43(7):e70329. doi: 10.1111/dme.70329 (PMC13257899; doi:10.1111/dme.70329)
Supplement: Supplementary file 3 — Appendix S1: [file DME-43-e70329-s002.docx]

**Appendix 1: Teplizumab Preparation and Administration**

Ancillary Equipment

- TZIELD® vials (teplizumab solution).
- 0.9% sodium chloride infusion bags (50 mL) for reconstitution and dilution.
- Sterile syringes (various sizes) and needles.
- Appropriate infusion delivery device - ideally the infusion line would not include a filter, but if a filter must be used the guidance is to use a PES filter
- Standard IV consumables: alcohol wipes, gauze, tape, tourniquet.
- Emergency medicines and resuscitation equipment for infusion reactions or Cytokine release syndrome (CRS).

Storage

- Store TZIELD® at 2–8 °C in original packaging until use.

Preparation

- Preparation must be performed using aseptic non-touch technique (ANTT) in accordance with local Standard Operating procedures (SOPs).
- Dilute according to manufacturer instructions:
  1. Prepare an intermediate dilution (e.g. 2 mL TZIELD® into 18 mL 0.9% sodium chloride) to achieve a 100 mcg/mL concentration in a total volume of 20mls. If the dose requires two vials of Teplizumab (e.g. on D5 if BSA is high), then two solutions should be prepared.
  2. Withdraw the calculated daily dose from the intermediate solution and add to a new 25mL 0.9% sodium chloride in infusion bag, syringe or glass vial.
  3. Mix gently; avoid shaking.
  4. Discard any unused intermediate solution in line with local policies
- Administer within 2 hours of preparation (maximum 4 hours at room temperature if not used immediately).

**Administration**

- Prime infusion line prior to administration
- Infuse over ≥30 minutes using an appropriate infusion delivery system.
- Flush the line with 25 mL 0.9% sodium chloride at the end of the infusion to ensure full delivery of the dose.
- Monitor patient for at least 1 hour post-infusion for adverse reactions.
- Blood monitoring should be performed on days 1, 3, 5, 8, and 14 before infusion; withhold or discontinue based on pre-defined safety parameters (see Table 2). More frequent monitoring may be warranted for any abnormalities, with continued surveillance until full resolution, as clinically indicated
